# Application of Random Forests Methods to Diabetic Retinopathy Classification Analyses

**DOI:** 10.1371/journal.pone.0098587

**Published:** 2014-06-18

**Authors:** Ramon Casanova, Santiago Saldana, Emily Y. Chew, Ronald P. Danis, Craig M. Greven, Walter T. Ambrosius

**Affiliations:** 1 Department of Biostatistical Sciences, Wake Forest School of Medicine, Winston-Salem, North Carolina, United States of America; 2 National Eye Institute, National Institutes of Health [NIH], Bethesda, Maryland, United States of America; 3 Fundus Photograph Reading Center, University of Wisconsin, Madison, Wisconsin, United States of America; 4 Wake Forest School of Medicine, Winston-Salem, North Carolina, United States of America; Zhongshan Ophthalmic Center, China

## Abstract

**Background:**

Diabetic retinopathy (DR) is one of the leading causes of blindness in the United States and world-wide. DR is a silent disease that may go unnoticed until it is too late for effective treatment. Therefore, early detection could improve the chances of therapeutic interventions that would alleviate its effects.

**Methodology:**

Graded fundus photography and systemic data from 3443 ACCORD-Eye Study participants were used to estimate Random Forest (RF) and logistic regression classifiers. We studied the impact of sample size on classifier performance and the possibility of using RF generated class conditional probabilities as metrics describing DR risk. RF measures of variable importance are used to detect factors that affect classification performance.

**Principal Findings:**

Both types of data were informative when discriminating participants with or without DR. RF based models produced much higher classification accuracy than those based on logistic regression. Combining both types of data did not increase accuracy but did increase statistical discrimination of healthy participants who subsequently did or did not have DR events during four years of follow-up. RF variable importance criteria revealed that microaneurysms counts in both eyes seemed to play the most important role in discrimination among the graded fundus variables, while the number of medicines and diabetes duration were the most relevant among the systemic variables.

**Conclusions and Significance:**

We have introduced RF methods to DR classification analyses based on fundus photography data. In addition, we propose an approach to DR risk assessment based on metrics derived from graded fundus photography and systemic data. Our results suggest that RF methods could be a valuable tool to diagnose DR diagnosis and evaluate its progression.

## Introduction

Diabetes results from the insufficient generation of insulin by the pancreas, or deficient insulin processing in the body. In 2011, the National Institutes of Health estimated there were 25.8 million people affected by diabetes in the US (8.3% of the population). Diabetic retinopathy (DR) is a common complication of diabetes; it affects more than 4.4 million people in the US aged 40 and older, and is one of the leading causes of blindness in the nation. Among those with diabetes, it is estimated that worldwide, approximately 93 million people may have some DR, and 28 million may have sight-threatening stages of DR [1]. Some of the major DR risks are considered to be duration of diabetes, blood pressure, glycemic control [2], [3], dyslipidemia [4], and nephropathy. Since not all diabetic patients develop DR some researchers believe genetic factors are involved [5].

DR is a silent disease that may not be detected until it is too late for effective treatment; therefore early detection could improve the chances of therapeutic interventions to alleviate its effects. Currently, DR detection is based on clinical examination or evaluation of digital color fundus photographs of the retina. These photographs are examined for evidence of lesions associated with DR, such as microaneurysms, hemorrhages, neovascularization or other vascular abnormalities and hard exudate deposits. Although this approach works well in general, the expertise needed to detect these problems is uncommon and intra- and inter-observer variability can affect the quality of the process [6]. Lack of expertise and equipment to diagnose DR is common, especially in rural areas or less developed countries. This has motivated increasing efforts to develop automated methods for DR detection using image processing, pattern recognition, and machine learning methods [6]–[8]. Most efforts have focused on creating automated systems that use the fundus photography images as input [9]–[12]. While good progress has been made and automated systems are beginning to reach standards similar to those of clinicians, the examination of fundus photography by experts still remains the gold standard.

Most previous machine learning research for potential use in DR is based on support vector machines (SVM) or other methods. Here we introduce Random Forests (RF) to DR classification analyses based on fundus photography data. RF is a powerful machine learning method for classification and regression which compares well with other state-of-the-art classifiers such as SVM [13] and ADABOOST [14]. The strengths of the RF approach are that: 1) it does not overfit; 2) it is robust to noise; 3) it has an internal mechanism to estimate error rates, called out-of-the-bag (OOB) error; 4) it provides indices of variable importance; 5) it naturally works with mixes of continuous and categorical variables; and 6) it can be used for data imputation and cluster analysis. These properties have made RF increasingly popular in the last few years, especially in the field of genetics and imaging [15]–[19]. In addition, rather than focusing on discriminating patients with DR from controls we proposed metrics for DR risk assessment based on Random Forests methods [20] using existing graded fundus photography and systemic data. The graded data from fundus photography can potentially contain subtle multivariate patterns predictive of early DR undetected by human experts. In this situation, the ability of high-dimensional machine learning algorithms to deal with multiple variables could be of great benefit.

The Action to Control Cardiovascular Risk in Diabetes (ACCORD) trial was designed to evaluate the effects of intensive versus standard interventions to control glucose, systolic blood pressure, and lipid levels on incidence of serious cardiovascular events in people with type 2 diabetes mellitus [21]–[23]. ACCORD-Eye was a substudy that sought to assess the effects of the interventions on retinal pathology at baseline and after 4 years in a subset of ACCORD participants [24]. The prospective study design, large sample size, baseline and follow-up fundus photography data, and the systematic record of eye events in ACCORD-Eye provided an opportunity to develop methods for early DR prediction.

We take advantage of our access to a well-characterized clinical database such as ACCORD-Eye to introduce RF to classification analyses of DR. We evaluated (a) its performance relative to logistic regression, a more conventional statistical approach, and (b) the impact of sample sizes on both classifiers. Finally, we have recently proposed the class-conditional probabilities generated by high-dimensional classifiers as a measure of risk for progression to Alzheimer’s disease (AD) [25]–[29]. Here we evaluate the use of class-conditional probabilities produced by RF to assess risk of future DR events in ACCORD-Eye participants. Our DR risk assessment metrics were derived from the fundus photography grading and systemic data obtained in the ACCORD study. These results could be a useful contribution for early detection of DR, and provide an interesting application for a highly valuable fundus photography database from over 3,400 individuals with diabetes mellitus.

## Materials and Methods

### ACCORD-eye Study

The design of the ACCORD-Eye study has been previously reported [24]. Briefly, in ACCORD, 10,251 middle-aged and elderly people with type 2 diabetes, hemoglobin A1C levels ≥7.5%, and additional cardiovascular disease risk factors were randomized to a glucose-lowering trial and either a blood pressure-lowering or fibrate trial. Cardiovascular events were ascertained every 4 months. ACCORD participants without a history of proliferative diabetic retinopathy treated with laser photocoagulation or vitrectomy also were eligible for the ACCORD Eye study. All ACCORD-Eye study participants provided written informed consent both for the overall ACCORD trial and the substudy. The ACCORD trial’s primary outcome was a composite comprising the first occurrence of a nonfatal myocardial infarction (MI), nonfatal stroke, or cardiovascular death. Secondary outcomes analyzed included total MIs and total strokes (i.e. fatal or nonfatal), cardiovascular death, and death from any cause. These study outcomes were adjudicated by investigators masked to treatment allocation. The mean follow-up period for the primary outcome and mortality were 4.7 years and 5.0 years, respectively.

The eye assessment consisted of comprehensive standardized eye examinations by a study ophthalmologist or optometrist, and fundus photography comprising seven standard stereoscopic fields obtained at baseline from 3433 subjects and at 4 years of follow-up available for 2856 participants. The fundus photographs were centrally graded by individuals masked to treatment allocation according to a modified version of the Early Treatment Diabetic Retinopathy Study (ETDRS) [30]. The severity of retinopathy at baseline and follow-up was classified as either no retinopathy; mild nonproliferative diabetic retinopathy (NPDR); moderate NPDR; or severe retinopathy (i.e. severe NPDR, proliferative retinopathy, or incident laser therapy or vitrectomy since baseline). Deterioration in diabetic retinopathy was classified as a <2-step, 2- to 3-step, or 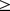
3-step change using the steps in the ETDRS person scale that evaluated both eyes. Anyone who had laser therapy or vitrectomy was deemed to have developed the most severe stage of diabetic retinopathy and was grouped with the >3-step change category. In this work, we will refer to changes from baseline leading to the 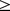
3-step change category as DR events. Information about the number of participants and DR events during follow-up in each DR severity group and follow-up diagnosis for the healthy participants at baseline is provided in Tables 1–2.

**Table 1:** Baseline stratification of subjects across DR severity groups and numbers of eye events per group is provided.

Groups	DR Event		All
	No Event	Event	
No DR	1536	92	1628
Mild DR	1331	84	1415
Moderate DR	201	23	224
Severe DR	114	52	166
All	3182	251	3433

DR events represent changes
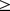
3 steps in the ETDRS scale during follow-up.

**Table 2:** Diagnosis after four years of follow-up for subjects without DR at baseline, and eye events for each subgroup.

Groups	DR Event		All
	No Event	Event	
No follow-up	253	13	266
No DR	531	29	560
Mild DR	606	39	645
Moderate DR	75	4	79
Severe DR	71	7	78
All	1536	92	1628

DR events represent changes
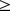
3 steps in the ETDRS scale during follow-up.

### Random Forests

RF is one of the so-called ensemble methods for classification, because a committee of learners (trees in this case) is generated and each one casts a vote for the predicted label of a given instance. The trees are built using the classification and regression trees methodology (CART) [31]. In constructing the ensemble of trees, RF uses two types of randomness: first, each tree is grown using a bootstrapped version of the training data. A second level of randomness is added when growing the tree by selecting a random sample of predictors at each node to choose the best split. The number of predictors selected at each node and the number of trees in the ensemble are the two main parameters of the RF algorithm. The RF developers have reported [20] that the method does not require much tuning of the parameters and the default values often produce good results for many problems. Once the forest is built, assigning a new instance to a class is accomplished by combining the trees, using a majority vote. As a result of using a bootstrap sampling of the training data, around one-third of the samples are omitted when building each tree. These are the so-called out-of-the-bag (OOB) samples that can be used to assess the performance of the classifier and to build measures of importance.

In this work, we used the permutation importance index to assess variable importance. The importance of a variable is evaluated by estimating the change in prediction error occurring when the variable in the OOB data is randomly permuted while others are left unchanged. The calculations are carried out tree by tree as the random forest is constructed. If a variable is important in a problem under analysis, permuting its values at random leads to larger changes in prediction performance compared to those that are unimportant. We used here the randomForest package in R [32] and its default parameters for RF: number of trees (ntree) equal to 500 and number of variables analyzed at each node to find the best split _G_ where 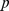 is the total number of variables in the problem.

Finally, we used RF capabilities for data imputation, which is based on the concept of proximities in RF. Proximities form a square matrix (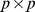) and in some sense are a measure of distance between two samples. To compute proximities after a tree is grown, all of the data, both training and OOB, are put down the tree. If two observations are in the same terminal node, their proximity is increased by one. At the end, the proximities are normalized by dividing by the number of trees. The proximity matrix is used to update the imputation of the missing values. For continuous predictors, the imputed value is the weighted average of the non-missing observations, where the weights are the proximities. For categorical predictors, the imputed value is the category with the largest average proximity. This process is iterated several times.

### Analyses

As variables we used measurements derived from fundus photography and systemic baseline data (see Tables S1 and S2) from the 3,433 ACCORD Eye participants. Follow-up data were available for 2856 participants. The data were formatted for analyses using the randomForest R library and the software package Weka [33]. Missing data were imputed using RF imputation methods [34]. Fields providing explicit information about DR severity were removed from the graded data.

For classification analyses we collapsed mild, moderate and severe DR groups into one class of subjects having DR which produced two classes: 1) those with no DR and 2) those with DR according to the graders assessments. Using baseline ACCORD-Eye data, we evaluated the accuracy of different classification models generated by combining (concatenating) fundus photography grading data with systemic variables. Specifically, we studied the value of the grading data (Eye data) and systemic data independently and combined for discriminating participants with and without DR, using both RF and the standard logistic regression. We selected logistic regression because: a) it is a conventional statistical method that models conditional probabilities, and b) it can highlight differences between RF and a traditional statistical method. The RF permutation index of variable importance was used to determine which variables play a major role when discriminating participants with and without DR. In addition, we studied the impact of the sample size on both algorithms’ performance. Sample sizes varied from 50 to 1700. In each case, 100 data training and testing datasets of the same size were drawn at random from the entire dataset. The models were estimated using the training datasets, while classification accuracy was estimated using the testing datasets, which is equivalent to a two-fold cross-validation. To evaluate the quality of the OOB RF mechanism, we calculated OOB accuracy rates to compare them with the results of the two-fold cross-validation. We also asked an expert to select a subset of eye variables with more clinical relevance (see Table S3). The analyses were rerun to study the impact of this selection on the performance of both methods.

Finally, we computed a RF model using data from all participants. The class-conditional probabilities of having DR were estimated at baseline for all participants who were diagnosed as not having DR and had follow up data. We then compared the probabilities of the subjects who later had eye events with the probabilities of those who did not, using the Wilcoxon rank sum test. Statistical testing was performed in SAS. The analyses were performed using probabilities computed for three different scenarios: 1) RF based on all variables; 2) RF based on ACCORD-Eye data only; and 3) RF based on systemic data only.

## Results

**Figure 1:**
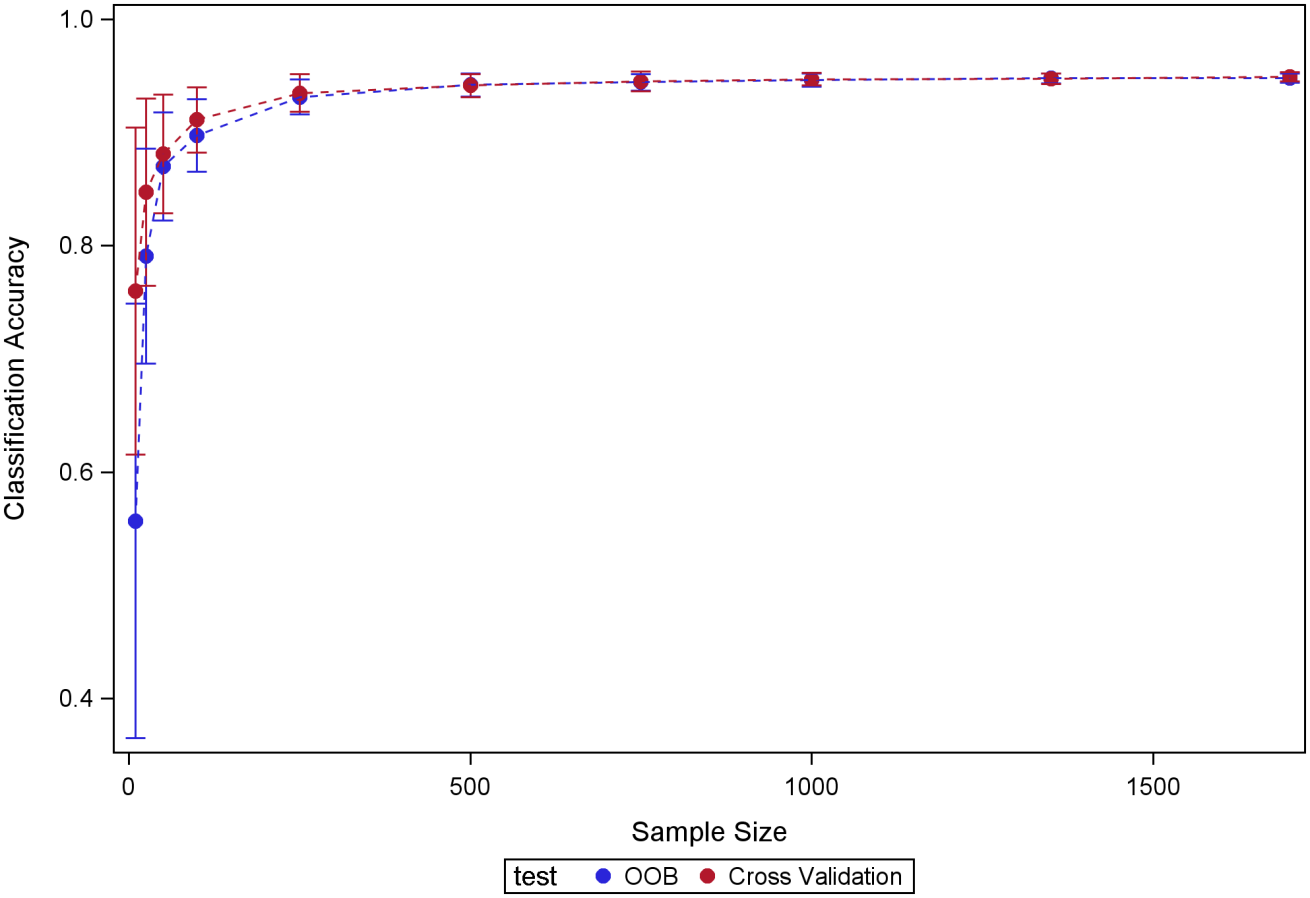
Estimates of RF classification accuracy obtained using the OOB mechanism and two-fold CV. RF models were estimated using all the available variables.

**Figure 2:**
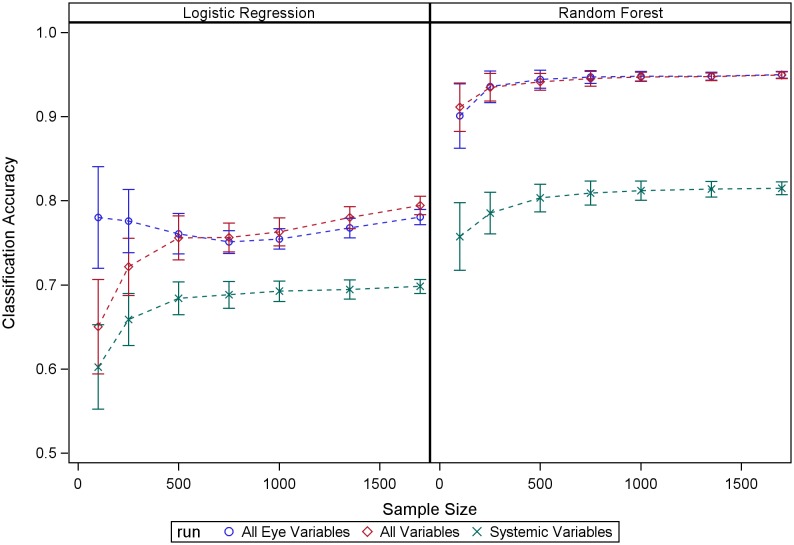
Performance across sample sizes of both RF (right panel) and LR is shown for three different scenarios: 1) Only eye data; 2) all variables in the study; and 3) only systemic data. The addition of systemic variables did not lead to significant increases in classification accuracy.

**Figure 3:**
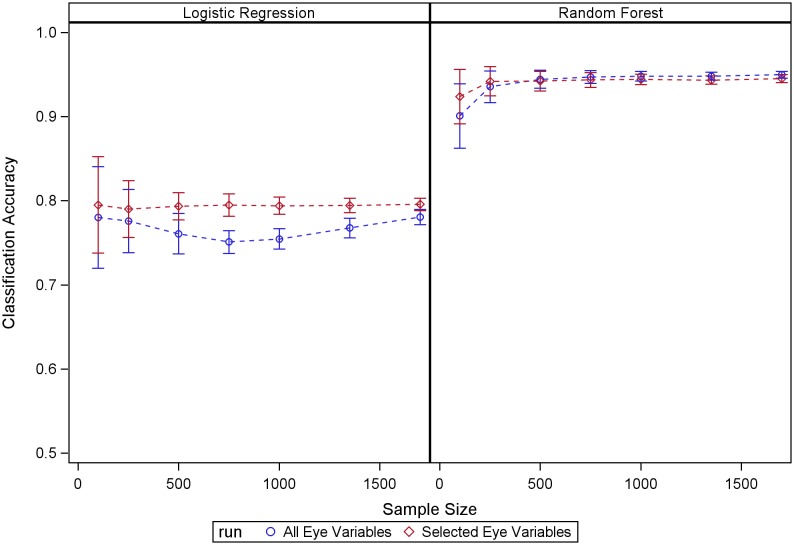
RF and LR performance using all available eye variables and a subset of eye variables selected by an expert as more clinically relevant. While this selection led to some improvements for LR it had very little impact on RF performance.

Figure 1 shows the RF classification accuracy estimated using the OOB mechanism built into RF, compared with the classification accuracy estimated using the testing datasets. Our results show that the RF OOB mechanism produces accurate estimates of RF performance within the evaluated sample sizes, suggesting reliable estimates of classifier performance for the full sample size in this study. RF outperformed LR in terms of classification accuracy across the three situations we studied (Figure 2). This advantage is very likely explained by RF nonlinearity and its capability to detect important variables in the model while discarding the effects of the non-relevant ones. Selecting a subset of clinical relevant variables in the graded fundus photography data based on an expert’s assessment led to moderate improvements in classification performance for logistic regression but had a much smaller impact on RF performance (see Figure 3). This experiment highlights RF robustness to the presence of large amount of non-relevant variables in the model.

Studies that report performance of classifiers’ performance across sample sizes are uncommon. We took advantage here of the size of the ACCORD-Eye dataset to evaluate the behavior of RF across sample sizes. Our experiments with fundus photography data provide empirical support for some reported RF properties such as the excellent quality of the estimates of accuracy provided by the OOB RF mechanism and its capability to discard the effect of non-relevant variables. The combination of graded fundus photography data with systemic variables in the model did not increase prediction performance, although systemic variables were informative regarding disease status (accuracy >75%). Finally, RF performance tended to stabilize at 500 samples. Further sample sizes increases produced very little or no gain in performance. On the other hand, LR-associated accuracies seemed to increase as the sample size increased.

This work provides insight into which variable might be most relevant to diagnose DR. The five most important variables for each scenario are presented in Table 3. RF measure of variable importance permutation index was used to rank the variables. Microaneurysm counts in both eyes were detected by RF as the most important variable for classification, both in the eye data only and in the combined data analyses. Number of medicines and diabetes duration also were important role in the analyses. Table 4 presents the average probabilities of having DR for two groups of participants who were not diagnosed with DR at baseline (a) participants who had a DR event (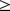
3 step ETDRS progression, vitrectomy, or laser photocoagulation) during the 4 years of follow up, and (b) participants who did not have a DR event. Participants who had the DR events were at a significantly higher risk of DR at baseline relative to those who did not have the DR events during follow-up. Although adding systemic data did not increase classification accuracy, combining both types of data lead to increased statistical discrimination of DR risk between those who did not have DR events during follow up and those who did.

**Table 3:** Most relevant variables according to RF permutation index criterion for each type of data.

Type of Data	Variables	Permutation Index (%)
Eye Only	Left microaneurysms count	53
	Right microaneurysms count	53
	Right abnormality2	41
	Left abnormality2	37
	Left Hard Exudate within grid	36
Systemic data	Number of medicines	60
	Diabetes duration	53
	ACCORD arm randomization	49
	Systolic blood pressure	28
	Body mass index	27
Combined	Left microaneurysms count	57
	Right microaneurysms count	57
	Number of medicines	48
	Right abnormality2	39
	Left abnormality2	38

The permutation index reflects decreases in classification performance when the values of a given variable have been randomly permuted. Abnormalities refer to the presence of different lesions detected by reviewers (e.g. drusens, age-related macular degeneration features, etc. - see Table S1). ACCORD arm randomization refers to membership to one of the eights arms of the ACCORD trial.

**Table 4:** The RF probabilities of having DR were estimated for two groups of participants who were not diagnosed as DR at baseline: a) those who had a DR event (> = 3 step ETDRS progression, vitrectomy, or laser photocoagulation) during follow-up and 2) those who did not.

DR event	Eye Data	Systemic data	Combined
No event mean (std)	0.06 (0.16)	0.34 (0.21)	0.15 (0.15)
Eventmean (std)	0.09 (0.18)	0.39 (0.21)	0.20 (0.17)
*p-value	0.03	0.01	0.0003

*Wilcoxon rank sum test, std – standard deviation.

Estimation was made using baseline data.

## Discussion

The main contribution of this work is the introduction of Random Forests methods to DR data analyses. Most previous work is based on the use of support vector machines (SVM) or other pattern recognition techniques. RF has shown great potential for DR classification and detection of relevant features in the fundus photography data. RF is highly nonlinear and works well with high-dimensional data clearly outperforming classic logistic regression. Another contribution is that while previous machine learning-based DR research has focused on classification of fundus photography images from patients with or without DR, here we have addressed the development of metrics to identify subjects at risk of DR in the very early stages. Our results suggest that it is possible to devise metrics that identify groups of participants at higher risk of DR. In the DR literature, only Rajendra and colleagues (using a very different rationale) have proposed an integrated index for DR identification based on images texture parameters [35]. Our work, on the other hand, is a translation of our Alzheimer’s disease (AD) research, where we have shown that similar metrics generated by classifiers estimated using neuroimaging and cognitive data are informative when discriminating between groups of subjects with mild cognitive impairment who will progress to AD from those who remain stable [28], [29], [36], [37]. Studies of the sample size effect on performance of machine learning algorithms are rare in the literature. Besides the mathematical interest of this question, this knowledge could save resources in clinical trials by reducing unnecessary computation when estimating prediction models.

Here, RF capabilities to detect relevant patterns in the data produced very meaningful results that correlate well with criteria for DR diagnosis and with known risk factors. For example, microaneurysms counts, microvascular abnormalities and hard exudates are important criteria used by clinicians to diagnose DR and evaluate its severity when using fundus photography data. Diabetes duration and blood pressure are widely recognized as major DR risk factors [1]. The number of medicines is an interesting finding. It could be an index of health status indicating that RF is detecting worsening in health associated with the DR group.

This work seeks to provide proof-of-concept for our methods in the area of DR prediction. However, our study does have some shortcomings. The main limitation is that our analyses were based on graded fundus photography data instead of the images themselves. To introduce into practice the approach proposed here, classification should be performed automatically using the fundus photographs replacing the grading by experts. We believe the quantification of subtle patterns in the images by pattern recognition and machine learning algorithms will help make these metrics much more accurate. Another possible source of improvement is using other types of information, such as genetic data, when available. Perhaps more sophisticated methods for data integration such as multiple kernel or manifold learning [38]–[40] will be more successful achieving synergy between different types of data than the concatenation of features we used. Another limitation is that our results can be confounded by misclassification errors by those who graded the images. This suggests an interesting application for our metrics that we will pursue in the future. Subjects declared as healthy by the graders but with high probability of having DR should raise a suspicion of misclassification error and those data should be reviewed by graders. This method could be a very useful tool for quality control in such large clinical trials as ACCORD-Eye. Finally, to have translational value, a validation study is needed using other databases.

We envision several very promising applications for the metrics proposed in this work that go beyond early detection. For example, they could be used to study DR genetics, which in general is poorly understood [41]. The genetic aspects of DR genetics are complex due to its relationship with glycemic control among other reasons. Our metrics could be used as quantitative traits in imaging genetic analyses of DR. We have recently reported that similar metrics derived from neuroimaging and cognitive data could increase statistical power in genome-wide association analyses of AD [27]. Recent research also provides growing evidence of association between small vessel disease and brain structure and function, suggesting the use of retinopathy as an earlier biomarker for brain small vessel disease [42]. A study published by the Women’s Health Initiative found associations between retinopathy, cognitive decline over 10 years, and ischemic lesion burden [43]. However, because retinopathy was relatively rare in that cohort, it was only evaluated as a dichotomized variable, limiting the ability to evaluate different degrees of retinopathy [42] – a situation where metrics such as those proposed here could be of great use. Finally, the metrics we have proposed here can be used to objectively select subjects at higher risk of DR for clinical trials if needed. This can spare resources by optimizing recruitment and decreasing required sample sizes.

## Conclusions

In this work, we have introduced an approach based on Random Forest methods to perform classification analyses of DR fundus photography data. RF clearly outperforms logistic regression, a conventional statistical approach, in most of the situations we evaluated. In addition, we generated metrics that assess risk of diabetic retinopathy, using graded fundus photography and systemic data. These metrics are sensitive to patterns in the data associated with future DR events in subjects not presently affected by DR. We will work towards refining these metrics by using other types of information and more sophisticated machine learning methods for multimodal data analyses.

## Supporting Information

Table S1 List of variables available from the fundus photography grading.
(DOCX)

Table S2 List of systemic variables.
(DOCX)

Table S3 Subset of clinical relevant eye variables according to expert criterion.
(DOCX)
