# Supplementary material for: Application of Random Forests Methods to Diabetic Retinopathy Classification Analyses
Source: PLoS One. 2014 Jun 18;9(6):e98587. doi: 10.1371/journal.pone.0098587 (PMC4062420; doi:10.1371/journal.pone.0098587)
Supplement: Table S1 — List of variables available from the fundus photography grading. (DOCX) [file pone.0098587.s001.docx]

Table S1 – List of variables available from the fundus photography grading.

| Field Description | Values |
| --- | --- |
| Camera type | 0 - Zeiss FF 2-4 (no notch)  [Zeiss classic 30° grid]  1 - Topcon 35° (square notch)  [Zeiss classic 30° grid]  2 - Kowa 30° (triangle at 12:00) or other  [Zeiss classic 30° grid]  3 - Zeiss FF 450 (triangle at 4:30)  [Zeiss FF 450 30° grid]  4 - Canon 40° (round notch)  [Canon 40° grid]  5 - Topcon 50° (square notch)  [Wide angle]  6 - Canon 60° (round notch)  [Wide angle]  7 - other  8 - cannot grade |
| Scatter (panretinal) photocoagulation | 0 - none  1 - questionable  2 - partial scatter or local PC for NV  3 - complete scatter (± local)  8 - cannot grade |
| Number of microaneurysms, required only of DRSeverity <= 20 | 0 - none  Q - questionable  1-10 (discrete count)  11 - 11+  88 - cannot grade |
| Hard exudate within grid, field 2 | 0 - none  1 - questionable  2 - definite  8 - cannot grade |
| Presence of retinal thickening | 0 - none  1 - questionable  2 - definite, outside grid  3 - definite, within grid  8 - cannot grade |
| Proximity of retinal thickening/adjacent hard exudate to the center in microns | 0, 100, 101, 200, 300, 301, 400, 500, 501, 600, 700, 800, 900, 1000...3000 - proximity in microns  8888 - cannot grade |
| Retinal thickening at center of macula | 0 - none  1 - questionable  2 - definite, < 1X reference  3 - definite, < 2X reference  4 - definite, ≥ 2X reference  8 - cannot grade |
| Clinically significant macular edema (ETDRS) | 0 - none  1 - questionable  2 - zone of RT ≥ 1 DA, part ≤ 1 DD from center  3 - RT or adjacent HE ≤ 500 μ from center  8 - cannot grade |
| Retinal thickening area within grid (center circle subfield) | 0 - 100% - retinal involvement  Q - questionable  CG - cannot grade |
| Retinal thickening area within grid (inner superior subfield) | 0 - 100% - retinal involvement  Q - questionable  CG - cannot grade |
| Retinal thickening area within grid (inner nasal subfield) | 0 - 100% - retinal involvement  Q - questionable  CG - cannot grade |
| Retinal thickening area within grid (inner inferior subfield) | 0 - 100% - retinal involvement  Q - questionable  CG - cannot grade |
| Retinal thickening area within grid (inner temporal subfield) | 0 - 100% - retinal involvement  Q - questionable  CG - cannot grade |
| Retinal thickening area within grid (outer superior subfield) | 0 - 100% - retinal involvement  Q - questionable  CG - cannot grade |
| Retinal thickening area within grid (outer nasal subfield) | 0 - 100% - retinal involvement  Q - questionable  CG - cannot grade |
| Retinal thickening area within grid (outer inferior subfield) | 0 - 100% - retinal involvement  Q - questionable  CG - cannot grade |
| Retinal thickening area within grid (outer temporal subfield) | 0 - 100% - retinal involvement  Q - questionable  CG - cannot grade |
| Hard exudate at center point (Pt) of grid | 0 - none  1 - questionable  2 - definite  8 - cannot grade |
| Hard exudate within grid (center circle subfield) | 0 - none  1 - questionable  2 - < circle C_0_  3 - < circle C_1_  4 - < circle C_2_  5 - < circle I_2_  6 - < circle O_2_  7 - > circle O_2_  8 - cannot grade |
| Hard exudate within grid (inner superior subfield) | 0 - none  1 - questionable  2 - < circle C_0_  3 - < circle C_1_  4 - < circle C_2_  5 - < circle I_2_  6 - < circle O_2_  7 - > circle O_2_  8 - cannot grade |
| Hard exudate within grid (inner nasal subfield) | 0 - none  1 - questionable  2 - < circle C_0_  3 - < circle C_1_  4 - < circle C_2_  5 - < circle I_2_  6 - < circle O_2_  7 - > circle O_2_  8 - cannot grade |
| Hard exudate within grid (inner inferior subfield) | 0 - none  1 - questionable  2 - < circle C_0_  3 - < circle C_1_  4 - < circle C_2_  5 - < circle I_2_  6 - < circle O_2_  7 - > circle O_2_  8 - cannot grade |
| Hard exudate within grid (inner temporal subfield) | 0 - none  1 - questionable  2 - < circle C_0_  3 - < circle C_1_  4 - < circle C_2_  5 - < circle I_2_  6 - < circle O_2_  7 - > circle O_2_  8 - cannot grade |
| Hard exudate within grid (outer superior subfield) | 0 - none  1 - questionable  2 - < circle C_0_  3 - < circle C_1_  4 - < circle C_2_  5 - < circle I_2_  6 - < circle O_2_  7 - > circle O_2_  8 - cannot grade |
| Hard exudate within grid (outer nasal subfield) | 0 - none  1 - questionable  2 - < circle C_0_  3 - < circle C_1_  4 - < circle C_2_  5 - < circle I_2_  6 - < circle O_2_  7 - > circle O_2_  8 - cannot grade |
| Hard exudate within grid (outer inferior subfield) | 0 - none  1 - questionable  2 - < circle C_0_  3 - < circle C_1_  4 - < circle C_2_  5 - < circle I_2_  6 - < circle O_2_  7 - > circle O_2_  8 - cannot grade |
| Hard exudate within grid (outer temporal subfield) | 0 - none  1 - questionable  2 - < circle C_0_  3 - < circle C_1_  4 - < circle C_2_  5 - < circle I_2_  6 - < circle O_2_  7 - > circle O_2_  8 - cannot grade |
| Confounding ocular abnormality likely to confound assessment of drug treatment effect or visual acuity measurement | 0 - none  1 - questionable  2 - present  3 - urgent condition confirmed by FPRC ophthalmologist  4 - possible adverse event confirmed by FPRC ophthalmologist  8 - cannot grade |
| Name of ocular abnormality 1 | 00 - large drusen area (> circle I_2_ in area)  01 - AMD RPE depigmentation  02 - AMD RPE hyperpigmentation  03 - AMD subretinal hemorrhage  04 - AMD subretinal fibrous scar  05 - AMD SSR/RPE detachment  06 - AMD geographic atrophy  07 - angioid streaks  08 - asteroid hyalosis  09 - central or branch artery occlusion  10 - central or branch vein occlusion  11 - chorioretinal scar: POHS, toxoplasmosis, other  12 - confounding anterior opacity (other than lens)  13 - drusen on the optic nervehead  14 - Hollenhorst plaque  15 - large cup/disc ratio  16 - confounding lens opacity  17 - macular hole or pseudohole  18 - nevus  19 - papillary swelling  20 - peripapillary atrophy, myopic crescent  21 - surface wrinkling / epiretinal membrane  22 - tension lines, dragged macula  23 - vitreous opacity or glial remnant  24 - other |
| Name of ocular abnormality 2 | [Same as Abnormality1] |
