# Supplementary material for: Application of Random Forests Methods to Diabetic Retinopathy Classification Analyses
Source: PLoS One. 2014 Jun 18;9(6):e98587. doi: 10.1371/journal.pone.0098587 (PMC4062420; doi:10.1371/journal.pone.0098587)
Supplement: Table S2 — List of systemic variables. (DOCX) [file pone.0098587.s002.docx]

Table S2 – List of systemic variables

| Diabetes duration |  |
| --- | --- |
| Randomized arm result. | 1='std glycemia/int BP'  2='std glycemia/std BP'  3='int glycemia/int BP'  4='int glycemia/std BP'  5='std glycemia/lipid agent'  6='std glycemia/lipid placebo'  7='int glycemia/lipid agent'  8='int glycemia/lipid placebo' |
| Dichotomized diabetes duration | 0=’diabetes duration <= 10 years’  1=’diabetes duration >= 10 years’ |
| Systolic Blood Pressure |  |
| UalbCreatinine Ratio |  |
| weight (kilograms) |  |
| Glycosylated hemoglobin percent |  |
| Trichotomized sbp | 0 = ‘sbp < 133’  1 = ‘133<=sbp<=144’  2 = ‘sbp > 144’ |
| Body Mass Index |  |
| triglycerides |  |
| diastolic blood pressure |  |
| Trichotomized visual acuity | Alpha = max(visacuityLeft,visacuityRight)  1 = alpha < 69  2 = 69 <= alpha < 84  3 = alpha >= 84 |
| Dichotomized bmi | 0 = ‘bmi < 30’  1 = ‘bmi >= 30’ |
| Visual Acuity |  |
| Trichotomized dbp | 0 = ‘dbp<72’  1 = ‘72<=dbp<=80’  2 = ‘dbp>80’ |
| Dichotomized age | 0 = ‘age<65’  1 = ‘age>=65’ |
| Visual Acuity Left (hxpe) |  |
|  |  |
| Dichotomized hba1c | 0 = ‘hba1c > 8.0’  1 = ‘hba1c <= 8.0’ |
| Visual Acuity Left (hxpe) |  |
|  |  |
| Dichotomized bmi | 0 = ‘bmi < 29’  1 = ‘bmi >= 29’ |
| High-density lipoprotein |  |
| Low-density lipoprotein |  |
| Compound variable consisting indicating high trigylcerides and low hdl | 0 = ‘trig < 204 and hdl > 34’  1 = ‘trig >= 204 and hdl <= 34’ |
| Trichotomized triglyceride score | 0 = ‘trig <= 128’  1 = ‘128 <trig <=203’  2 = ‘trig > 203’ |
| Randomization variable based on patient id | 0 = ‘default case’  1 = ‘if patient id starts with 1’ |
| Trichotomized ldl | 0 = ‘ldl <=84’  1 = ’84 < ldl <= 111’  2 = ‘ldl > 111’ |
| Randomization variable | Based on first number of patid |
| Smoking status | 0 = ‘never smoked’  1 = ‘former smoker’  2 = ‘current smoker’ |
| Race field | 0 = ‘white’  1 = ‘nonwhite, including PacificIslander,asian,black, FirstNation, AmericanIndian, OtherRace and Spanish’ |
| Trichotomized hdl | 0 = ‘hdl <= 34’  1 = ’34 < hdl <= 40’  2 = ‘hdl > 40’ |
| sex | 0 = ‘male’  1 = ‘female’ |
| Dichotomous smoking status | 0 = ‘smoked’  1 = ‘never smoked’ |
| Dichotomized smoking field (identical to neversmoker) | 0 = ‘smoked’  1 = ‘never smoked’ |
| Randomization variable based on patient id | 0 = ‘default case’  1 = ‘if patient id starts with 2’ |
| Dichotomized smoking field (identical to neversmoker) | 0 = ‘never smoked’  1 = ‘formerly smoked’ |
| Race field | 0 = ‘white,nonwhite, including PacificIslander,asian,black, FirstNation, AmericanIndian, OtherRace and Spanish’  1 = ‘other race’ |
| Dichotomized smoking field (identical to neversmoker) | 0 = ‘not currently smoking’  1 = ‘currently smoking’ |
| Race Field | 0 = ‘Not American Indian’  1 = ‘AmericanIndian’ |
| Race Field | 0 = ‘Not PacificIslander’  1 = ‘PacificIslander’ |
| Race Field | 0 = ‘Not Asian’  1 = ‘Asian’ |
