# Supplementary material for: Application of Random Forests Methods to Diabetic Retinopathy Classification Analyses
Source: PLoS One. 2014 Jun 18;9(6):e98587. doi: 10.1371/journal.pone.0098587 (PMC4062420; doi:10.1371/journal.pone.0098587)
Supplement: Table S3 — Subset of clinical relevant eye variables according to expert criterion. (DOCX) [file pone.0098587.s003.docx]

Table S3 – Subset of clinical relevant eye variables according to expert criterion.

| **Variables Description** |
| --- |
| Number of microaneurysms, |
| Hard exudate within grid, field 2 |
| Presence of retinal thickening |
| Proximity of retinal thickening/adjacent hard exudate to the center in microns |
| Retinal thickening at center of macula |
| Clinically significant macular edema (ETDRS) |
| Hard exudate at center point (Pt) of grid |
